# Clival Screw Placement in Patient with atlas assimilation: A CT-based feasibility study

**DOI:** 10.1038/srep31648

**Published:** 2016-08-19

**Authors:** Wei Ji, Xiang Liu, Wenhan Huang, Zucheng Huang, Jianting Chen, Qingan Zhu, Zenghui Wu

**Affiliations:** 1 Department of Spinal Surgery, Nanfang Hospital, Southern Medical University, Guangzhou, China; 2 Hospital of Orthopaedics, Guangzhou General Hospital of Guangzhou Military Command (Liuhuaqiao Hospital), 111 Liuhua Road, Guangzhou, China

## Abstract

Clival screw and plate fixation technique is an alternative or supplement to the occipitocervical instrumentation. However, no report has clarified the applied anatomy of clivus in patients with atlas assimilation (C1A), especially for clival screw fixation. Therefore, we measured the parameters of clival lengths, widths, putative screw lengths, clival-cervical angel and foramen magnum diameters on CT images in a cohort of 81 C1A patients and patients without C1A. The clivus showed a rectangular shape in 96.3% (78/81) of C1A patients, and a normal-like triangle shape in 3.7% (3/81) of C1A patients. The intracranial clival length decreased 13% (37 mm) in C1A patients, the extracranial clival length 14.8% (24.1 mm), the clival-cervical angle 6.2% (122.3°) and the superior screw length 11.3% (14.1 mm), the sagittal diameter of foramen magnum 16% (28.0 mm), respectively. There was no significant difference in the widest or narrowest clival width, or the middle screw length, or the transverse diameter of foramen magnum between groups. The inferior clivus was feasible for an average 9.7-mm-length screw placement in C1A patients, while not in patients without C1A. The present study characterizes clivus of C1A patients with an unnormal-like rectangular shape, and confirmes a screw placement at the inferior clivus.

The C1 assimilation (C1A) is defined as partial or complete congenital fusion of the occiput and atlas, which is caused by failure of segmentation between the fourth occipital and the first cervical sclerotome during embryonic development^1^,^2^,^3^, and the reported prevalence is ranged from 0.08% to 2.76%^4^,^5^ in the general population without gender difference. The C1A is always associated with basilar invagination and atlantoaxial dislocation, resulting in compression of the cervicomedullary by the odontoid process^6^. Neurologic deterioration in the C1A patients commonly occurs at the third or fourth decade of life, and requires a surgical treatment^7^,^8^.

To successfully stabilize the mechanically compromised occipitocervical junction, one must surgically correct deformity and decompress the neural structures. Occipitocervical fixation was widely used in disorders related with instability of the occipitocervical junciton^9^,^10^,^11^,^12^. Alternatively, anterior occipitocervical fusion using the clivus as the cephalad anchoring point was reported in several studies^13^,^14^,^15^. For a chordoma patient underwent a single-stage total C-2 intralesional spondylectomy, Suchomel *et al*.^13^ conducted a stability reconstruction of the occipitocervical junction by using a fashioned mesh cage with screws purchasing to the clivus cranially and C3 vertebral body caudally (Fig. 1).

Recently, the present authors evaluated the feasibility and optimal trajectory for clival screw fixation^16^,^17^,^18^. However, in patients with C1A, the morphology and volume of C1 and C2 are significantly altered^19^,^20^,^21^. Interestingly, we found that the clival morphology and volume of patients with C1A was also significantly different from the normal one (Fig. 2). There has been no literature on the applied anatomy of clival screw purchase for C1A patients. Therefore, we hypothesis the C1A a relative constant morphology in the craniocervical junction, and design a CT-based morphometric study of clivus in C1A patients. The objective of the present study was to identify morphometric difference between patients with and without C1A, evaluate the feasibility and risk of clival screw placement, and establish guidelines for clival screw fixation in C1A patients.

## Material and Methods

This was a retrospective analysis of patients of East Asian ancestry who presented to the department of orthopedics in our hospital between January 2001 and June 2015, requiring CT scanning of the head and cervical spine. A total of 81 patients with C1A were available for this study, with age of 44.4 ± 12.8 years old. Ethical approval and written consents from the participants were waived due to the retrospective design of the present study. However, their personal information were anonymized and de-identified before analysis. Two experienced spinal surgeons reviewed the CT scans of the patients and looked for C1A and other congenital malformations of the craniovertebral region such as basilar invagination, Klippel-Feil syndrome, Chiari malformation, and vertebral artery anomaly. The diagnosis of C1A was assessed on sagittal and coronal CT images. CT images were taken using a General Electric CT scanner (Philips Brilliance 16 CT; Philips Medical Systems, Eindhoven, The Netherlands) with slice thickness of 1 mm, pitch of 0.7 mm, 120 kV, 180 mA, 512 × 512 matrix, and reconstruction level of 1 mm. Images of the sagittal and axial planes of the craniovertebral region were obtained after multiplanar reconstruction on the workstation (MXV, Philips). According to the measurement procedure of our previous study^17^, the intracranial and extracranial clival lengths, clival-cervical angle, and projected screw lengths were measured in the midsagittal plane, and the clival widest and narrowest diameters, and the diameters of the foramen magnum in the axial planes.

**Figure 3:**
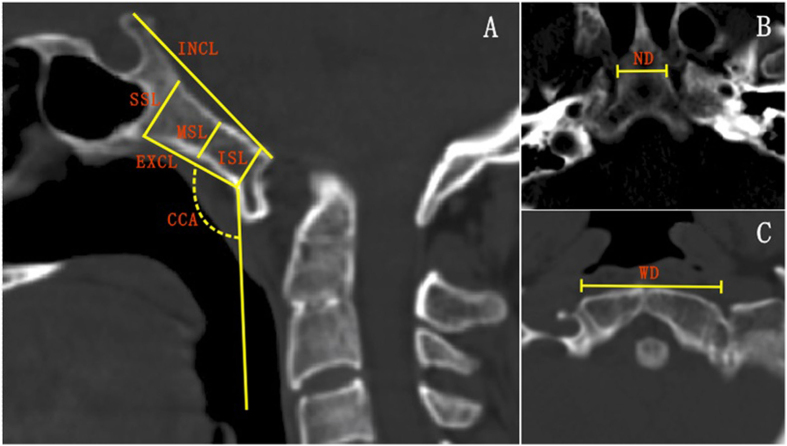
Measurements of clivus on CT images. (**A**) The intracranial clival length (INCL), extracranial clival length (EXCL), superior screw length (SSL), middle screw length (MSL) and inferior screw length (ISL), and clival-cervical angle (CCA) were illustrated on the midsagittal image of the craniovertebral region. (**B**) The narrowest diameter of clivus (ND). (**C**) The widest diameter of clivus (WD) were defined on an axial image.

The intracranial clival length was defined as the distance from the level of the dorsum sellae to the anterior margin of the great occipital foramen, and the extracranial clival length the maximal distance between the bottom and top portions of the extracranial clivus in the midsagittal plane (Fig. 3A). The superior, middle and inferior clival screw entrances were located at the superior, middle and inferior points of the extracranial clivus, respectively, and along the line perpendicular to the tangent of the extracranial clivus in the midsagittal plane (Fig. 3A). The putative superior, middle and inferior screws were simulated by a cylinder of 3.5 mm in diameter. The clival-cervical angle was the angle between the tangent of the extracranial clivus and the tangent of the anterior cervical vertebrae in the midsagittal plane. The widest diameter was defined as the distance between the anterolateral left and right occipital condyles (Fig. 3B), while the narrowest diameter was the width of the cross sections through the top portion of the extracranial clivus (Fig. 3C).

**Figure 4:**
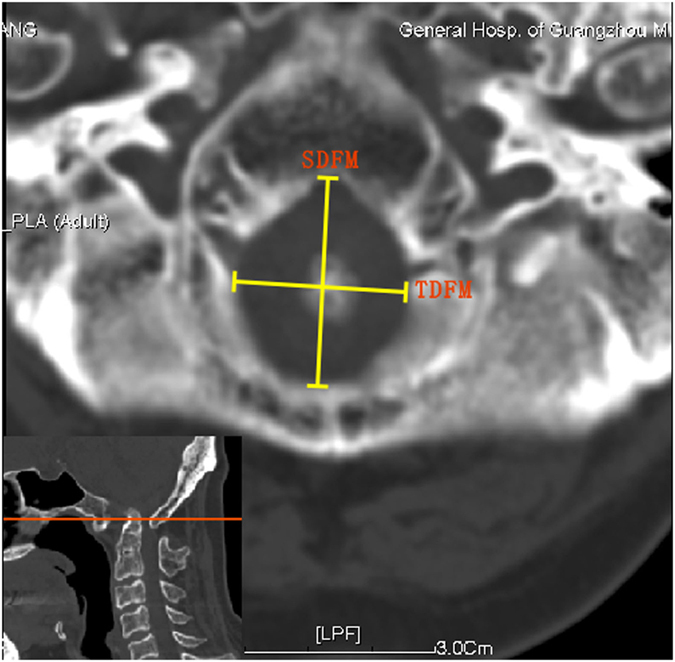
Diameter measurements of foramen magnum on CT images. SDFM, sagittal diameter of foramen magnum; TDFM, transverse diameter of foramen magnum.

The diameters of the foramen magnum were measured in the sections across the foramen magnum (Fig. 4). The sagittal diameter of the foramen magnum (SDFM) was defined as the greatest anteroposterior dimension of the foramen magnum, and the transverse diameter of the foramen magnum (TDFM) as the greatest width of the foramen magnum.

Patients without C1A matched to the C1A patient cohort in age and gender were randomly selected from the same database as a control. The aforementioned measurements were applied for the control group.

Two observers (W. J. and X. L.) performed CT morphometric measurements. The intraobserver and interobserver reliability were calculated. Each parameter was re-measured in 1 set of 15 patients randomly selected at 3-week intervals, and the intra-class correlation coefficient was calculated.

### Statistical analyses

The statistical analyses were carried out using SPSS (SPSS Inc, Chicago, IL). The normality of data distribution was screened with Shapiro-Wilk test. Independent-Samples t-test was used to compare measurements between genders and groups. Measured putative screw lengths were compared within groups using repeated-measures one-way analysis of variance test with post hoc Tukey tests for individual comparisons. Statistical significance was set at *P* < 0.05.

## Results

### General data

A total of 81 patients with C1A (44.4 ± 12.8 years old with a range from 19 to 73 years) were available for this study, including 34 (42%) male and 47 (58%) female patients. The control group was selected to math the C1A patient cohort in age and gender, respectively. There are 96.3% (78/81) of C1A patients with rectangular-in-shape clivus, and 3.7% (3/81) with triangle-in-shape clivus. All patients without C1A has triangle-in-shape clivus.

### Clival lengths

**Figure 5:**
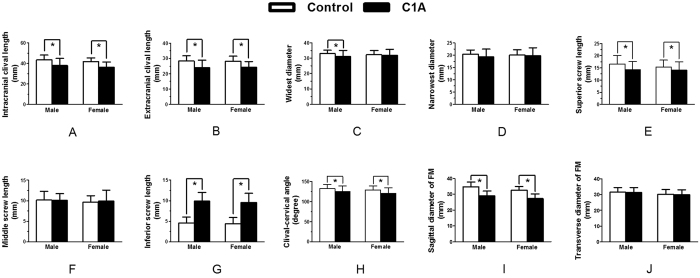
Measurements of clivus and FM in patients with and without C1 assimilation based on gender. *p < 0.05. FM, foramen magnum.

Both the intracranial and extracranial clival lengths were smaller in the C1A group than the control group (37.0 ± 5.8 mm vs. 42.5 ± 4.1 mm, p < 0.001; 24.1 ± 4.2 mm vs. 28.3 ± 3.3 mm, p < 0.001). The intracranial and extracranial clival lengths decreased 13% and 15% in patients with C1A, respectively. These changes were significant in male and female patients, respectively (p < 0.001) (Fig. 5A,B).

### Clival widths

There was no significant difference in the widest or narrowest clival width between the C1A and control groups (31.6 ± 4.0 mm vs. 32.5 ± 2.6 mm, p = 0.075; 19.6 ± 3.2 mm vs. 20.2 ± 2.0 mm, p = 0.187). The only significant difference was observed in the clival widest diameter of between male patients with and without C1A (p = 0.026) (Fig. 5C,D).

### Clival putative screw lengths

**Figure 6:**
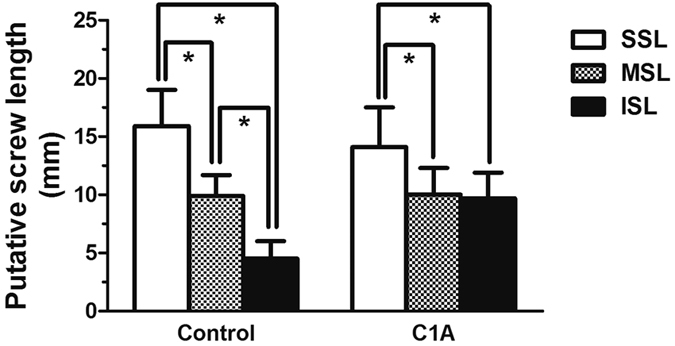
Clival screw lengths in patients with and without C1 assimilation. *p < 0.05.

The superior screw length was shorter in the C1A group than the control group (14.1 ± 3.4 mm vs. 15.9 ± 3.1 mm, p = 0.001), but the inferior screw length was longer in the C1A group than in the control group (9.7 ± 2.2 mm vs. 4.5 ± 1.5 mm, p < 0.001). These significant differences were also observed in the male and female subgroups (Fig. 5E–G). Note of that the inferior screw length increased 116% in the C1A group. No significant difference was found in the middle screw length between groups (10.0 ± 2.3 mm vs. 9.9 ± 1.8 mm, p = 0.759). The putative screw lengths of patients without C1A were significantly different among the superior, middle and inferior screw lengths, while the putative screw length of patients with C1A was only significantly longer in the superior screw length (Fig. 6).

### Clival-cervical angle

The clival-cervical angle was smaller in the C1A group than in the control group (123° ± 14° vs. 130° ± 10°, p < 0.001). These significant differences were also observed in the male and female subgroups (Fig. 5H).

### Foramen magnum diameters

The sagittal diameter of the foramen magnum was smaller in the C1A group than the control group (28.0 ± 3.1 mm vs. 33.4 ± 3.0 mm, p < 0.001). These significant differences were also observed in the male and female subgroups (Fig. 5I). No significantly difference was found in the transverse diameter of the foramen magnum between groups (30.6 ± 3.1 mm vs. 30.8 ± 3.0 mm, p = 0.625) or subgroups of male or female patients (Fig. 5J).

The intra-observer reliability ranged from 0.90 to 0.96 for the initial examiner and from 0.90 to 0.95 for the secondary observer. The inter-observer reliability ranged from 0.87 to 0.93.

## Discussion

Although the etiology remains unclear, it is commonly observed multiple bony and soft tissue anomalies in C1A patients^22^. Therefore, morphological data from general population may not apply for C1A patients. The present study showed that the clival axial length and putative screw length at superior clivus were smaller in C1A patients, as well as the clival-cervial angle. Interestingly, the putative screw length at the inferior clivus was more than double length in C1A patients, and similar to the putative screw length at the middle clivus. Therefore, longer screw purchase at the inferior clivus is feasible to C1A patients, decreasing dissection or exposure of the clivus.

The C1A is commonly associated with basilar invagination and atlantoaxial dislocation, and makes the odontoid process compress the cervicomedullary^6^ and requires a surgical interventionton to stabilize the occipitocervical junction, to correct the deformity and decompress the neural structures^7^,^8^. Occipitocervical fixation spanning from occiput to C2 has been used to treat the instability of occipitocervical junction^9^,^10^,^11^,^12^. However, C1 and C2 screw placement is more difficult and dangerous in C1A patients. The C1 lateral mass and condyle are hypoplastic and fused together in the C1A patients, resulting in obvious changes in morphology of C1 lateral mass^19^ and difficulty for screw insertion in the lateral mass^20^. Furthermore, Aoyama *et al*.^21^ carried out a radiographic measurement of C1A patients and demonstrated that the C2 bony structures were significantly smaller than the normal, making the C2 pedicle screw or translaminar screw placement more difficult. In addition, the vertebral artery of C1A patients sometimes made the surgical dissection and mobilization extremely difficult^19^,^22^,^23^. Therefore, the abnormal osseous anatomy and higher risk of injury to the vascular and neurologic structures preclude a posterior occipitocervical fixation in C1A patients.

Anterior instrumentation anchored to the clivus is an alternative or supplement to the posterior occipitocervical constructs. In 1969, Deandra*d*e *et al*.^24^ reported an anterior cervical fixation from the skull base to C3 after laminectomy from C1 to C3 for a patient with rheumatoid arthritis. Goel *et al*.^15^ also developed an anterior plate fixation with proximal screws anchoring to the clivus of a 12-year-old boy with congenital basilar invagination, avoiding the secondary posterior fixation. Furthermore, Suchomel *et al*.^13^ performed a single-stage total C-2 intralesional spondylectomy for a chordoma patient and reconstructed the anterior defect using a 14-mm diameter Harms mesh cage filled with autologous bone graft, and secured the cage to the clivus and C3 body with screws. Rawlins *et al*.^14^ further replace the mesh cage with a plate. The present authors determined the clival screw purchase and designed a novel clivus plate for craniovertebral instability^16^,^17^,^18^.

The intracranial clival length of patients without C1A in the present study was 43 mm and within the range (40–45 mm) reported in literatures^25^,^26^, while the extracranial clival length, the clival widest and narrowest diameters were consistent with previous studies^16^,^17^,^26^. The clival-cervical angle of patients with C1A in the present study was also smaller than that in our precious study (122° vs. 130°)^16^,^17^.

The clivus is surrounded by the dura, basilar artery, pons, ventral medulla oblongata and the V~XII cranial nerves^27^. On the extracranical clivus, pharyngeal tubercle (around 15 mm) can be a central mark to guide a safe dissection of the soft structures and exposure to the clivus^16^. Nevertheless, the clival length in patients with C1A was 4 mm shorter than general population (24.2 mm vs. 28.1 mm), rendering less operative field and more meticulous technique for screw placement.

Usually, the clivus is slightly concave in longitudinal and transversal directions. According to the our previous study^16^,^17^, the ideal screw entry points were at the middle part of the clivus. The upper clivus runs from the dorsum sellae and posterior sphenoid sinus to the plane of dorellos canal. The sphenoid sinus may extend backwards below the hypopyseal fossa and sometimes into the occipital bone^28^, causing the clival screw inserted into the sphenoid sinus or the pituitary fossa. In addition, the upper screw placement also requires more exposure. On the other hand, there is less thickness and a shorter screw length at the inferior clivus. However, the inferior clivus was thick in C1A patients and feasible to a screw placement. The inferior screw length was close to the middle screw length in C1A patients. It is expected that the screw entrances were lower, and close to the foramen magnum in C1A patients with lesser exposure to the clivus. Therefore, the clival screw fixation technique is feasible for C1A patients.

Size of the foramen is critical parameter for the manifestation of clinical signs and symptoms in craniocervical pathology, such as motor myelopathy, sensory abnormalities, brainstem and lower cranial nerve dysfunctions, etc^29^. Diseases associated with anomalies of the foramen magnum include occipital vertebra, basilar invagination, condylar hypoplasia, and atlas assimilation^30^. Interestingly, the sagittal and transverse diameters of the foramen magnum were independent risk factors in those patients needing craniocervical decompression^31^,^32^,^33^.

In the present study, the sagittal diameters of the foramen magnum were about 37.2 mm in males and 34.6 mm in females, and the transverse diameters of the foramen magnum were 31.6 mm in males and 29.3 mm in females^34^, respectively. These results were higher than the sagittal diameter (34.9 mm in males and 32.9 mm in females) and the transverse diameter (29.5 mm in males and 27.3 mm in females) reported by Uthman *et al*.^35^. Catalina-Herrera^36^ reported the sagittal diameter of the foramen magnum was 35.2 mm and transverse diameter of the foramen magnum was 30.3 mm, while Schmeltzer^37^ reported the sagittal diameter was 35 mm and the transverse diameter was 30 mm. The diameters of the foramen magnum of the control group in the present study was comparable to data reported in literatures. However, the data from the C1A group in the present study are smaller. Therefore, this bony abnormalities resulting in changes of the anatomy of the foramen magnum would be benefit to the decision-making process for the diagnosis and treatment of such disorders.

Several anterior approaches have been proposed for the clivus and upper cervical spine, such as the transoral, the transoral-transpalatopharyngeal, the transmandibular and the high anterior cervical retropharyngeal approaches^38^,^39^,^40^. The clival-cervical angle is smaller in patients with C1A patients, and results in the clival screw placement more difficult or impossible through the transoral approach alone. Suchomel *et al*.^13^ performed C2 spondylectomy through a combined transoral approach and created a small right-sided submandibular channel through the floor of the mouth in order to achieve a straight angle to the clival screws. Rawlins *et al*.^14^ reached the access to the anterior upper cervical spine and clivus through mandibular osteotomy and soft palate split. Additionally, the endoscopic approach to the clivus may become an alternative approache since more exposure of clivus is achievable with angled endoscopes^27^.

In this study, we have a good sample size for radiographic measurements in C1A patients, although the prevalence of occipitalization in the general population ranges only from 0.08% to 2.76%^4^,^5^. The measurements were performed using radiograph images rather than directly on cadaveric specimens. Nevertheless, previous reports have demonstrated measurements obtained from CT significantly correlated with the actual anatomic measurements and established the guidelines for screw placement^41^.

## Conclusion

This study, in the first time, identified the morphometric characters of the clivus in C1A patients, and suggested a lower clival screw placement feasible. Our results suggested that the inferior clivus in patients with C1A was thick enough for a screw purchase, and for a screw placement at the middle-inferior clivus less surgical dissection and exposure.

## Additional Information

**How to cite this article**: Ji, W. *et al*. Clival Screw Placement in Patient with atlas assimilation: A CT-based feasibility study. *Sci. Rep.* **6**, 31648; doi: 10.1038/srep31648 (2016).

## Figures and Tables

**Figure 1:**
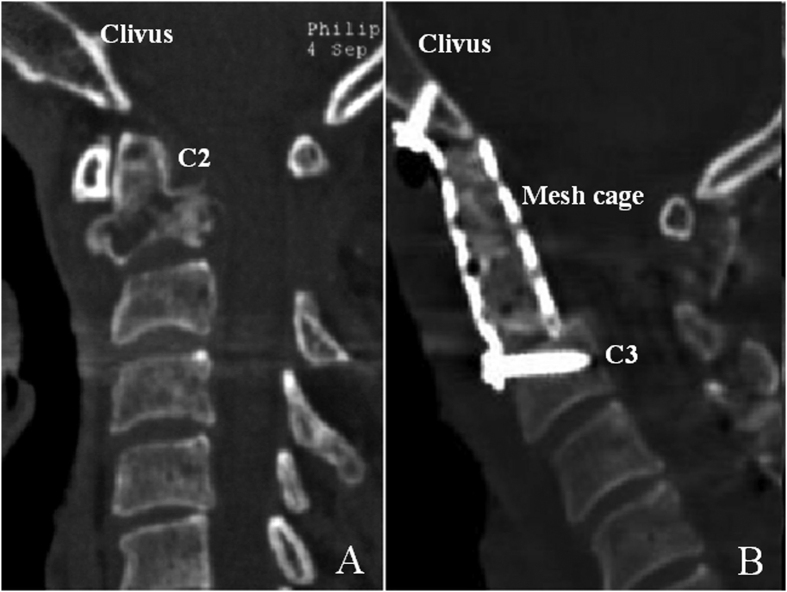
Preoperative sagittal (**A**) reconstructed CT images of the cervical spine with an osteolytic destruction of C-2; Underwent a single-stage total C-2 intralesional spondylectomy, a postoperative sagittal (**B**) reconstructed CT images of the cervical spine showing the mesh cage screwed into the clivus cranially and C3 vertebral body caudally^13^. C2, the second cervical vertebra; C3, the third cervical vertebra.

**Figure 2:**
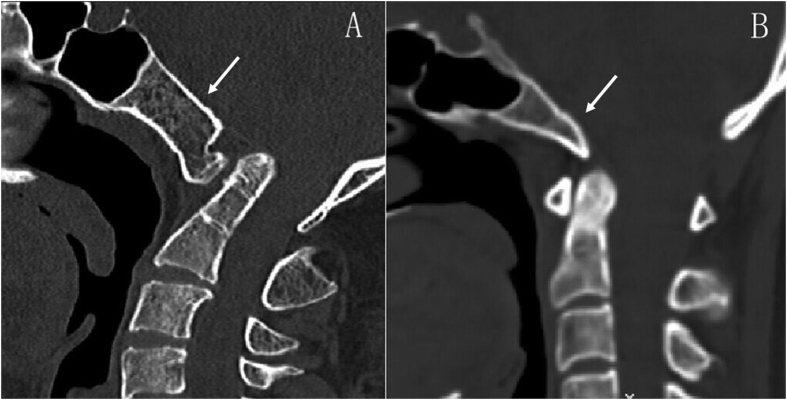
The clivus (white arrows) shown on the sagittal CT images in patients with (**A**) and without (**B**) C1 assimilation.
